# MIL-68 (Ga) for the extraction of derivatized and non-derivatized parabens from healthcare products

**DOI:** 10.1038/s41598-023-48880-1

**Published:** 2023-12-02

**Authors:** Sakha Pezhhanfar, Mir Ali Farajzadeh, Mahdi Kheirkhah Ghaleh, Seyed Abolfazl Hosseini-Yazdi, Mohammad Reza Afshar Mogaddam

**Affiliations:** 1 Department of Analytical Chemistry, Faculty of Chemistry, University of Tabriz, Tabriz, Iran; 2 Engineering Faculty, Near East University, Mersin 10, 99138 Nicosia, North Cyprus Turkey; 3 Department of Inorganic Chemistry, Faculty of Chemistry, University of Tabriz, Tabriz, Iran; 4 Food and Drug Safety Research Center, Tabriz University of Medical Sciences, Tabriz, Iran; 5 Pharmaceutical Analysis Research Center, Tabriz University of Medical Sciences, Tabriz, Iran

**Keywords:** Environmental sciences, Chemistry

## Abstract

This study was the first-ever attempt to apply MIL-68 (Ga) in developing an analytical method. The method extracts and preconcentrates some parabens from mouthwash and hydrating gel samples. The variable extraction parameters were optimized, and the figures of merit were documented. Avogadro software was used besides discussing intermolecular interactions to clarify the absorption process. ComplexGAPI software was also exploited to assess the greenness of the method. After the derivatization of the parabens using acetic anhydride in the presence of sodium carbonate, sodium chloride was added to the solution and vortexed to dissolve. A few milligrams of MIL-68 (Ga) were added into the solution and vortexed. Centrifugation separated the analyte-loaded absorbent, which was treated with mL volume of methanol through vortexing for desorption aim. A few microliters of 1,2-dibromoethane were merged with the methanolic phase and injected into a sodium chloride solution. One microliter of the extracted phase was injected into a gas chromatograph equipped with a flame ionization detector. High enrichment factors (200–330), reasonable extraction recoveries (40–66%), wide linear ranges (265–30,000 µg L^−1^), and appreciable coefficients of determination (0.996–0.999) were documented. The applicability of dispersive solid phase extraction for extracting polar analytes, imposing no additional step for performing derivatization, the capability of MIL-68 (Ga) for the absorption of both derivatized and non-derivatized parabens, the use of only 10 mg absorbent, and one-pot synthesis besides no high temperature or long reaction time in the sorbent provision are the highlights of the method.

## Introduction

Parabens such as methylparaben (Mep), ethylparaben (Etp), and propylparaben (Prp) (*p*-hydroxybenzoic acid esters) are used to preserve pharmaceuticals, foods, and cosmetics^1^. Their application has become widespread because of antimicrobial and antifungal activities, solubility in aqueous and organic solvents, low cost, no taste and odor, activity in a wide pH range, and stability in different matrices^2,3^. Despite their preservative functions, they adversely affect animal and human health. Their detection in human breast tumors^4,5^ and placental tissues^6^ has raised concerns. Parabens can also disrupt or modulate the endocrine system^7^. It was observed that parabens are associated with gestational diabetes^8^. The decrease and increase in the weights of the thyroid and adrenal glands, respectively, have also been documented as the side effects of parabens^9^. Decreasing the sperm quality of male rats exposed to parabens has been reported^10^. So, detecting and determining parabens in samples that are in direct contact with humans is of great importance. Until now, ultra-performance liquid chromatography^11^, high-performance liquid chromatography^12^, high-performance thin-layer chromatography^13^, and gas chromatography (GC)^14^ have been used for parabens’ monitoring. Directly injecting the matrices containing parabens into the analytical apparatuses is rarely possible due to the samples' complexity, viscosity, and high matrix effect. So, a sample preparation step is needed to result in low limits of detection (LODs) and quantification (LOQs). Moreover, in the case of parabens, a derivatization step can avoid peak tailing besides increasing the peak area and height for the same concentration of the analyte. The consequences decrease the LOD and LOQ values. Up until now, some derivatization approaches including diazomethane- and acetic anhydride-based alkylation^15,16^, bis (trimethylsilyl) trifluoroacetamide- and N-methyl-N-(*tert*-butyldimethylsilyl) trifluoroacetamide-based silylation^17,18^, and silylation with fluoroacetyl groups^19^ and silyl^20^ have been developed. So far, some extraction methods such as dispersive liquid–liquid microextraction (DLLME)^21^, magnetically-assisted matrix solid phase dispersion extraction^22^, cloud point extraction^23^, micro solid phase extractio^24^, microdisk solid phase extraction^25^, stir bar sorptive extraction^26^, ultrasound-assisted solid-phase extraction^27^, and single drop microextraction (SDME)^28^ have been developed for the extraction of parabens from different samples. There are some limitations such as the high matrix effect in DLLME, instability of the drop in SDME, and low extraction recovery (ER) and enrichment factor (EF) values when applying solid phase approaches since parabens are polar compounds and have high solubility values in aqueous media. To overcome the limitations, metal–organic frameworks (MOFs) can be applied as a member of coordination polymers that are hybrid crystalline compounds, and more than 50% of their crystal volume is occupied by porosity^29,30^. They benefit from diversity in dimension and the type of ligand and cation sections that result in modifiability, proper surface area, storage capacity, chemical and thermal stability due to strong bonds, and specific pore volume and diameter^31,32^. Based on the mentioned features, MOFs have gained attention in the fields of hydrogen storage^33^, water treatment^34^, extraction procedures^35,36^, and supercapacitors^37^. Up to now, some MOFs and MOF composites such as chitosan@UiO-66 (Zr)^38^, polydopamine-coated MIL101@Fe_3_O_4_@ carrageenan hydrogel beads^39^, HKUST-1^40^, Cr-MOF^41^, MIL-101 (Cr)^42^, and Fe_3_O_4_@multiwalled carbon nanotubes@MIL-101 (Cr)^43^ have been used for the extraction of parabens. Although dispersive solid phase extraction (DSPE) of parabens using MOFs seems beneficial, applying dispersive micro solid phase extraction (DµSPE) precedes DSPE by using low MOF weights which is more economical^44^. The problematic aspect of DµSPE is the low EF values. So, combining DµSPE and DLLME increases the EFs and reduces the matrix effect^45^.

In this study, *for the first time*, a gallium-based MOF named MIL-68 (Ga) was applied in the field of analytical method development for the extraction and preconcentration of parabens from mouthwash and hydrating gel samples. Since parabens are polar compounds, their extraction through a solid phase microextraction procedure is promising, whereas most of the extraction processes for parabens are based on liquid phase microextraction approaches. Moreover, MIL-68 (Ga) showed appreciable capability to absorb both *derivatized* and *non-derivatized* parabens. So, the derivatization step can be done before the absorption or after the desorption step. This observation illustrates the absorptive ability of the MOF towards the specific target compounds according to the discussed mechanism in section "The extraction mechanism of parabens using MIL-68 (Ga)". The developed MOF-based DµSPE process could extract parabens from some complex samples such as gel matrices. The adopted efficient and simple derivatization process which was done in the DµSPE section without any additional step led to the betterment of the chromatographic behavior of the analytes by increasing the peak height and area, and avoiding peak tailing. Also, it resulted in lowering the LODs and LOQs of the analytes. Moreover, the derivatization step decreased the solubility of the analytes in the aqueous phase leading to increasing ERs. DµSPE reduced the matrix effect of the samples, and DLLME increased the EF values. The extraction was propelled by using a low weight of the MOF. The subjection of MIL-68 (Ga) to methanol in the desorption step led to puffing up the MOF particles proving its porosity and favorable desorption ability. Only a microliter amount of the organic extraction solvent was used in the study. Moreover, the one-pot synthesis process and the low temperature and short time for providing the MOF are appreciable. Mouthwash and hydrating gel samples were subjected to MIL-68 (Ga)-based DµSPE-DLLME procedure and the final extracted phase was introduced into a GC-flame ionization detection (FID).

## Materials and methods

### Chemicals and solutions

The applied ligand, gallium salt, and solvent for the synthesis of MIL-68 (Ga) including 1,4-benzenedicarboxylic acid (1,4-BDCA), gallium nitrate nonahydrate, and *N*,*N*-dimethylformamide (DMF), respectively, were purchased from Merck (Darmstadt, Germany). Sodium chloride and sodium sulfate as the salting-out agents of DµSPE and DLLME sections were provided by Merck. The extraction solvents used in DLLME including carbon tetrachloride, 1,2-dibromoethane (1,2-DBE), and 1,1,1-trichloroethane (1,1,1-TCE) (analytical grade) were bought from Janssen (Beerse, Belgium). Sodium carbonate, as a base for the facilitation of the derivatization procedure, acetic anhydride, as the derivatization agent, and the applied disperser solvents including 2-propanol, acetonitrile (ACN), and methanol (analytical grade) were from Merck. The surveyed analytes including Mep, Etp, and Prp were from Sigma (St. Louis, MO, USA). Deionized water was bought from Ghazi Co. (Tabriz, Iran). It was used to prepare the solutions used in DµSPE and DLLME. Hydrochloric acid (37%, *w/w*) and sodium hydroxide were purchased from Merck and used in pH adjustments. A 5000 mg L^−1^ stock solution of the compounds of interest was prepared in methanol and used for the preparation of the working aqueous solutions by dilution.

### Samples

Paraben-free stated solutions including three mouthwash and three hydrating gel samples were purchased from local pharmacies of Tabriz (East Azerbaijan Province, Iran). They were stored at room temperature before being extracted by the developed method. The mouthwash and hydrating gel samples were diluted at 1:4 and 0.5:4.5 (*v/v*) ratios, respectively, with deionized water.

### Apparatus

The separation of Mep, Etp, and Prp was done using a Shimadzu gas chromatograph (2014, Kyoto, Japan) with an FID and a splitless/split injection port. The column oven temperature was fixed at 40 °C for 1 min to focus the injected phase at the beginning of the column and help to separate the analytes and decrease peak tailing. Then, the temperature was increased to 300 °C at the rate of 20 °C min^−1^. The temperature ramp led to the separation of the parabens which resulted in a short run time. Eventually, the temperature was maintained at 300 °C for 1 min. Zebron capillary column (5% diphenyl, 95% dimethyl polysiloxane; Phenomenex, Torrance, CA, the USA), (30 m × 0.25 mm i.d., with a film thickness of 0.25 μm) was used for the separation of the analytes. Helium (99.999%; Gulf Cryo, Dubai, United Arab Emirates) was used as the makeup (flow rate, 30 mL min^−1^) and carrier (linear velocity, 30 cm s^−1^) gasses. The temperature of 300 °C was fixed for both the FID and injection port. The sampling time and split ratio of the injection port were 1 min and 1:10, respectively. The air inlet of FID was set at 300 mL min^−1^. The fuel (hydrogen) at the flow rate of 30 mL min^−1^ was generated by a Shimadzu hydrogen generator (OPGU-1500S). A Metrohm pH meter (Herisau, Switzerland), model 654, was utilized in the pH adjustment of the solutions. A Hettich centrifuge (D-7200, Kirchlengern, Germany) was used in the DµSPE and DLLME steps. For the dispersion of MIL-68 (Ga) into the solutions to facilitate the absorption process, an L46 vortex (Labinco, Breda, the Netherlands) was used. A UT 12 Heraeus oven (Hanau, Germany) was used in the synthesis process of the MOF. Different analyses including Brunauer–Emmett–Teller (BET, BELSORP-mini II, Japan) for obtaining the surface area, total pore volume, and average pore diameter; scanning electron microscopy (SEM) (Mira 3 microscope, Tescan, Czech Republic) for recording the morphology of the MOF; energy dispersive X-ray (EDX) for the surface elemental analysis; Fourier transform infrared (FTIR) spectrophotometry (Bruker, Billerica, USA) for investigating the functional groups; and X-ray diffraction (XRD) (Siemens D500 diffractometer, Siemens AG, Karlsruhe, Germany) for crystallinity evaluation were carried out on MIL-68 (Ga).

### Synthesis of MIL-68 (Ga)

MIL-68 (Ga) was synthesized by upscaling a previously documented process introduced by Volkringer et al.^46^. For the MOF provision, 2 g of gallium nitrate nonahydrate was dissolved in 15.75 mL of DMF. Also, 0.85 g of 1,4-BDCA was dissolved in 30 mL of DMF. The solutions were stirred for 15 min. Then, both solutions were transferred into a Teflon-lined stainless steel autoclave and heated in an oven set at 100 °C for 10 h. A white precipitate was obtained as the result of the synthesis process. The MOF was filtered using filter paper and washed with 40 mL of DMF (four times, each time with 10 mL). After drying at room temperature overnight, MIL-68 (Ga) was placed in a beaker and located in an oven set at 150 °C for 8 h. The weight of the final product was 1.38 g. The characterization analyses were conducted on the obtained MOF. MIL-68 (Ga) was subjected to the developed method for the extraction and preconcentration of parabens.

### Standard solution of derivatized parabens

Since the direct injection of the standard solution of parabens results in peak tailing, 10 µL of 5000 mg L^−1^ stock solution (with respect to each analyte) and 10 µL of acetic anhydride were mixed and vortexed for 1 min to perform the derivatization. The obtained 2500 mg L^−1^ (with respect to each analyte) derivatized standard solution was injected into the separation system three times each day to monitor the quality of the separation system. The obtained peak areas were used to calculate the ERs and EFs of the analytes.

### Extraction procedure

#### DμSPE

**Figure 1:**
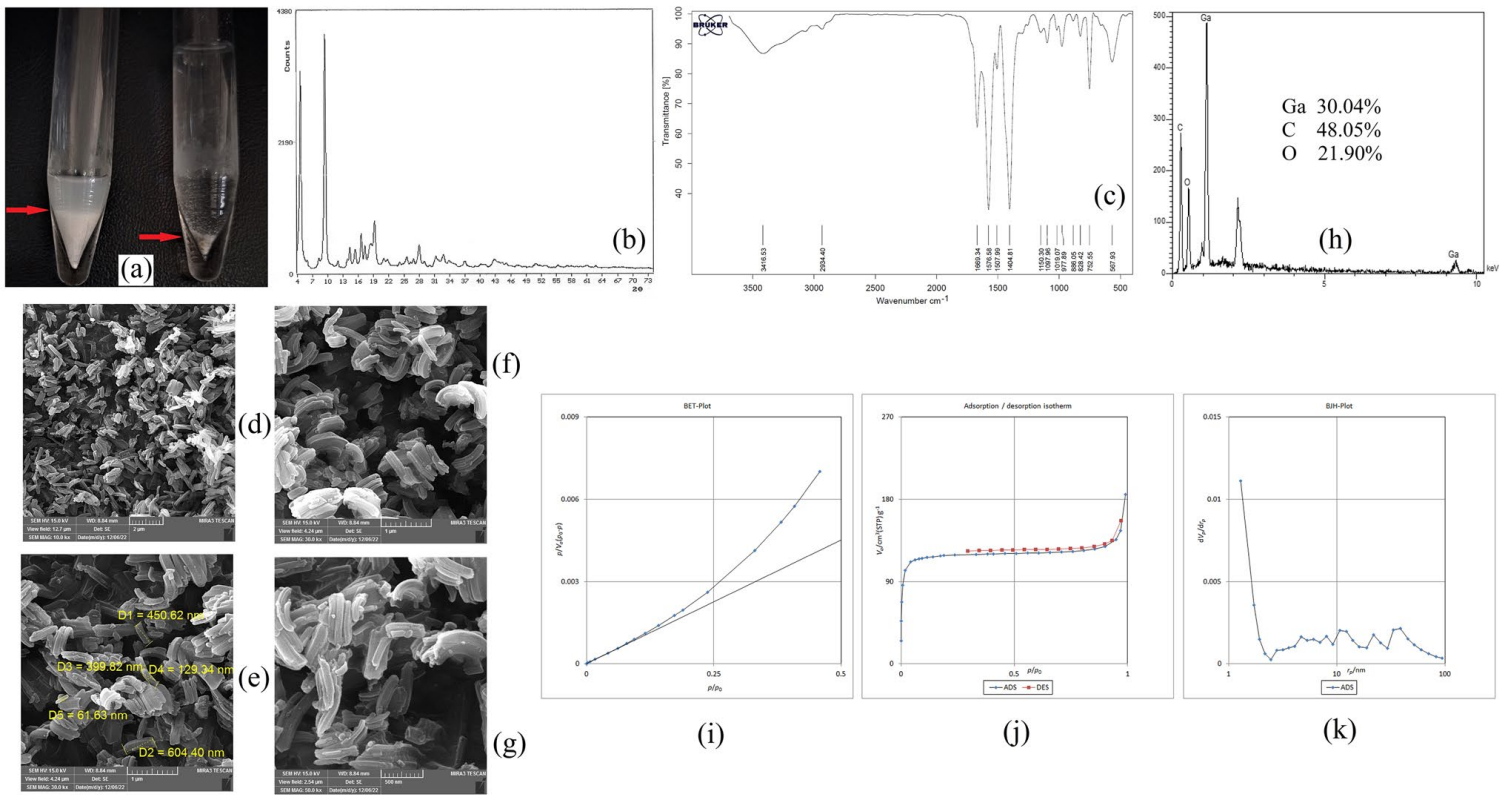
MOF particles in the aqueous (right) and methanolic solutions (left) (**a**), XRD pattern (**b**), FTIR spectrum (**c**), SEM images (**d**, **e**, **f**, **g**), EDX spectrum (**h**), BET plot (**i**), nitrogen adsorption/desorption isotherm (**j**), and BJH plot (**k**) of MIL-68 (Ga).

To 5 mL of deionized water, located in a 10-mL conical bottom glass test tube, 5 mg L^−1^ of each surveyed paraben was spiked. 100 mg of sodium carbonate was added into the solution and vortexed to dissolve. Then, 100 µL of acetic anhydride was added and vortexed for 1 min. After the accomplishment of the derivatization step, 250 mg of sodium chloride was added to the solution and vortexed. MIL-68 (Ga) (10 mg) was added into the solution and vortexed for 5 min to absorb the compounds of interest. Then, the suspension was subjected to centrifugation for 5 min at 5000 rpm. After discarding the supernatant, the paraben-loaded MOF was treated with 1 mL of methanol by vortexing for 5 min. Figure 1a (the right one) shows 10 mg of MIL-68 (Ga) added into the initial 5-mL aqueous solution in the absorption step, and the left one illustrates the same weight of the MOF after vortexing in methanol. It is seen that the MOF particles are puffed up and the occupied volume at the bottom of the test tube has dramatically increased. This is favorable since it can lead to the proper desorption of the analytes from MIL-68 (Ga). Subsequently, centrifugation was done for 5 min at 7000 rpm to separate the methanolic solution from the MOF particles. The analyte-containing methanolic solution was subjected to the following preconcentration step (DLLME).

#### DLLME

To the obtained organic phase from the previous section, 25 µL of 1,2-DBE was added, and the solution was sucked into a 5-mL glass syringe. A 5 mL aqueous solution having 500 mg of dissolved sodium chloride was prepared and the organic content of the syringe was swiftly injected into the aqueous solution. As a result, a cloudy solution appeared which was the outcome of creating the tiny droplets of 1,2-DBE dispersed into the aqueous solution. Eventually, centrifugation was implemented for 5 min at 5000 rpm to sediment the extracted phase (10 ± 0.5 µL). One microliter of the sedimented phase was injected into the separation system.

### EF and ER calculations

The performed preconcentration on the analytes through the method is shown by EF. This term illustrates the ratio of the organic phase analyte concentration (C_org_) to the analyte’s concentration in the aqueous phase (C_0_). Equation (1) shows EF calculation.

1 $$EF=\frac{{C}_{org}}{{C}_{0}}$$

The percentage of the migrated analyte into the extracted phase is shown by ER. Based on Eq. (2) it is understood that the percentage of the migrated analyte number into the organic phase (n_fin_) to the same term in the aqueous solution (n_0_) multiplied by 100 is called ER.

2 $$ER=\frac{{n}_{fin}}{{n}_{0}}\times 100=\frac{{C}_{fin\times {V}_{fin}}}{{C}_{0} \times {V}_{aq}}\times 100=EF\times \frac{{V}_{fin}}{{V}_{ aq}}\times 100$$

In this equation, V_aq_ is the volume of the initial aqueous phase and V_fin_ is the volume of the organic phase.

## Results and discussion

### Characterization of MIL-68 (Ga)

To obtain the XRD pattern of the synthesized MOF and also to infer that MIL-68 (Ga) has been obtained carefully, XRD analysis was carried out. In the XRD analysis, the crystallographic planes of a chemical compound compose the XRD pattern. This study was performed in the 2*θ* range of 4–74°. It can be seen from Fig. 1b that there are explicit peaks with different intensities. Some XRD peaks at the approximate 2*θ* values of 5, 9, 14, 15, 17, 18, 19, and 28° are vivid according to the obtained pattern. Observing distinctive peaks proves the crystallinity of the synthesized phase. Furthermore, the excellent overlapping between the recorded XRD pattern in the previous study^46^ and the one obtained in this research proves the successful provision of the target MOF.

Figure 1c demonstrates the FTIR spectrum of MIL-68 (Ga) in the wavenumber range of 400–4000 cm^−1^. The FTIR spectrum of 1,4-BDCA is provided in the literature^47^. As can be seen, various sharp FTIR peaks emerged that represent the functional groups of the hybrid compound. The absorption peak at 1669.34 cm^−1^ shows C=O stretching of carboxylate groups. The absorption peaks at 1576.58, 1507.99, and 1404.81 cm^−1^ show asymmetric and symmetric stretching of carboxylate groups. The absorption peaks at 1150.30, 1097.96, and 1019.07 cm^−1^ demonstrate the C-O stretching bonds that are the basis of the gallium-to-oxygen bonds created through the deprotonation of the hydroxyl groups. C=C bending which stems from the cyclic section of the MOF’s ligand is seen from 977.89, 886.05, 828.42, and 752.55 cm^−1^ absorption peaks. The absorption peak at 567.93 cm^−1^ can be attributed to the attachment of gallium to oxygen in the structure of MIL-68 (Ga).

The SEM image of a chemical compound provides a visual understanding of its morphology, shape, and size distribution. Figure 1d–f, and g demonstrate the obtained SEM images for MIL-68 (Ga) in the magnification scales of 10,000, 30,000, 30,000, and 50,000 times, respectively. An electron beam of 15,000 V was applied to obtain the documented images. The work distance to provide the images was 8.84 mm in all cases. The images show the rod-like individual particles of MIL-68 (Ga) that seem to be bent next to each other. Figure 1d demonstrates the particles of MIL-68 (Ga) with a wider view field (12.7 µm). This figure shows that the particles are piled up in different directions. According to Fig. 1e, the longitudinal and transverse dimensions are in the ranges of 399–604 and 61–129 nm, respectively. The smallness of the particles can facilitate the absorption procedure.

EDX analysis was also carried out on MIL-68 (Ga). EDX results indicate the percentage ratio of the surface elements in a compound. Figure 1h shows the EDX outcome of the MOF. It is seen that the percentages of gallium, carbon, and oxygen are 30.04, 48.05, and 21.90%, respectively. The peak at around 2 keV position is related to gold that was used when preparing the sample for the analysis. No extra elemental peak is seen that denotes the MOF purity.

BET analysis results in beneficial data based on nitrogen absorption/desorption experiments. The obtained BET plot for MIL-68 (Ga) is seen in Fig. 1i. Also, the nitrogen adsorption–desorption isotherm and BJH (Barrett-Joyner-Halenda) diagrams are shown in Fig. 1j,k, respectively. 482.87 m^2^ g^−1^ surface area, 0.2839 cm^3^ g^−1^ total pore volume, and 2.3517 nm average pore diameter are the obtained data for the synthesized MOF. A high surface area and small pore diameter (in the semi-microporous scale) are the features of the prepared MOF.

### Optimization of variable parameters

**Figure 2:**
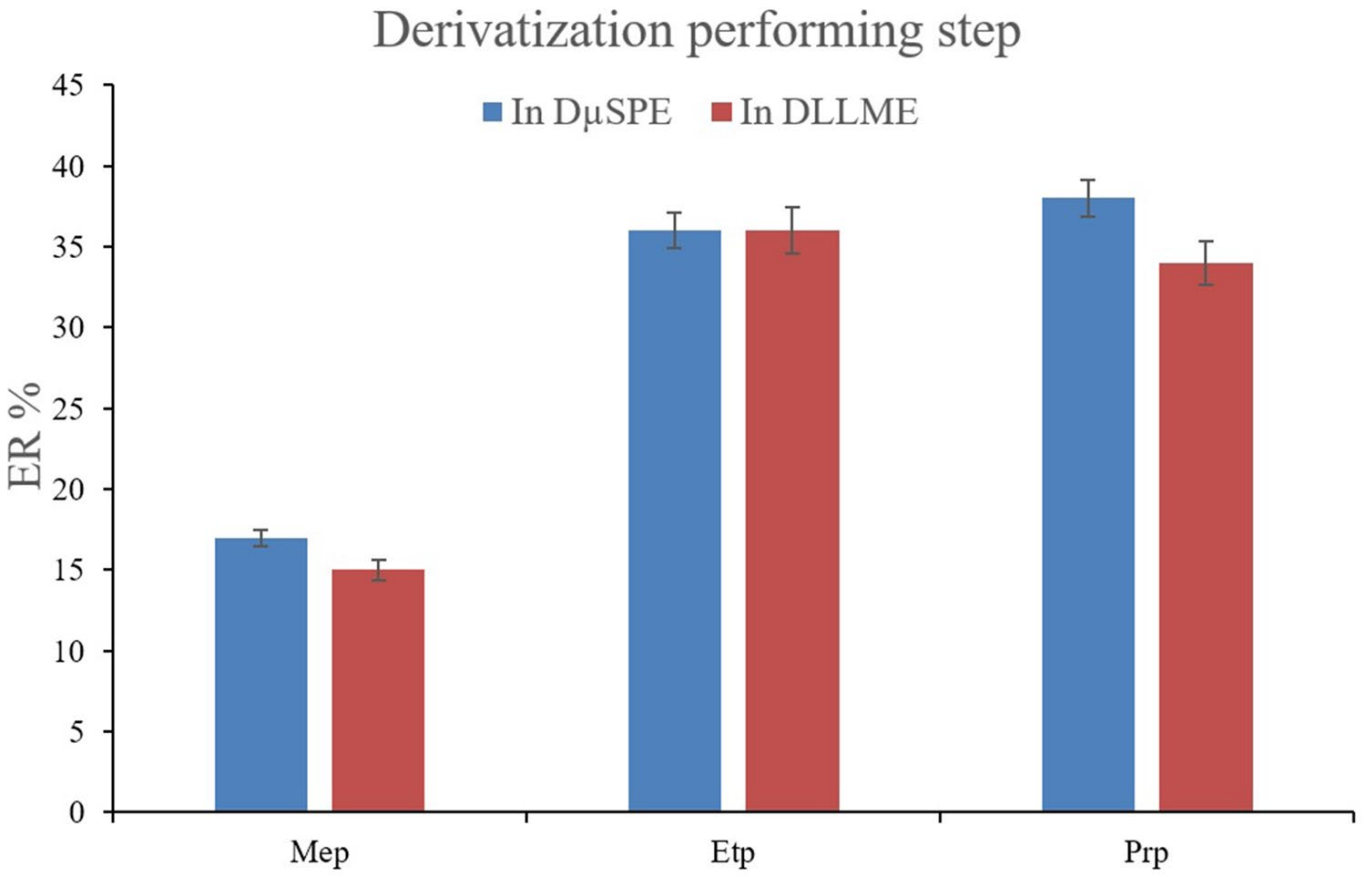
Selection of derivatization performing step. Extraction conditions: DµSPE procedure: aqueous sample volume, 5 mL deionized water spiked with 10 mg L^−1^ of each analyte having 100 mg dissolved sodium carbonate and 100 µL acetic anhydride; vortex time in absorption step, 5 min; desorption solvent (volume), ACN (1.0 mL); vortex time in desorption step, 5 min; and centrifugation speed and time, 6000 rpm and 5 min, respectively. DLLME procedure: aqueous phase, 5 mL deionized water without salt addition and pH adjustment; extraction solvent (volume), 1,2-DBE (30 and 28 µL in the cases of pre- and post-absorption derivatization); centrifugation rate, 6000 rpm; and centrifugation time, 5 min. The error bars show the minimum and maximum of three repeated determinations.

To reach the highest efficiency of the method for the extraction of the parabens from aqueous media, some important variables that can affect the ERs of the analytes were optimized. The first parameter that underwent optimization was to assess the impact of pre- and post-absorption derivatization. In this step, the surveyed parabens were derivatized in the DµSPE and DLLME sections separately. Derivatization in DµSPE led to the absorption of derivatized parabens by the MOF. On the other hand, non-derivatized parabens were absorbed by MIL-68 (Ga) in the post-absorption derivatization approach. So, the derivatization procedure was performed after the absorption step and in the DLLME solution. In the case of pre-absorption derivatization, the aqueous solution of DµSPE was enriched with 100 mg of dissolved sodium carbonate after spiking the analytes. Then, 100 µL of acetic anhydride was added and the solution was vortexed. Consequently, the MOF was added into the solution and the extraction process proceeded. In the case of post-absorption derivatization, following the absorption of the parabens by the MOF, 100 µL of acetic anhydride was added into the methanolic solution after the desorption process and injected into the 5-mL aqueous solution of DLLME having 100 mg of dissolved sodium carbonate. Figure 2 shows the outcome of the experiments. It is seen that although there is no dramatic difference between the obtained ERs by applying the two approaches, the ERs related to the derivatization approach in DµSPE are slightly better in the cases of Mep and Prp. This is a promising point for the extraction procedure that proves the capability and high affinity of MIL-68 (Ga) for the absorption of derivatized and non-derivatized Mep, Etp, and Prp from aqueous solutions. Moreover, performing the derivatization process in DLLME leads to an unstable sedimented extracted phase and its suction into the injection syringe is challenging. The phenomenon stems from the fact that the produced carbonic acid in the solution due to the presence of sodium carbonate and acetic acid produced by acetic anhydride generates carbon dioxide gas. Entering the injection syringe into the solution leads to the release of the gas and makes the extracted phase unstable. It is worth mentioning that proper chromatographic peaks were obtained in both cases. 30 and 28 µL of 1,2-DBE were used to obtain 10 ± 0.5 µL of the final extracted phase in DµSPE and DLLME derivatization steps. The observation denotes the successful derivatization of the analytes in both approaches. According to the obtained data and the discussed observations, the derivatization approach in DµSPE was chosen to be applied. Applying the derivatization step in the DµSPE step has another benefit that enables the usability of the method in the approaches that DLLME is omitted.

**Figure 3:**
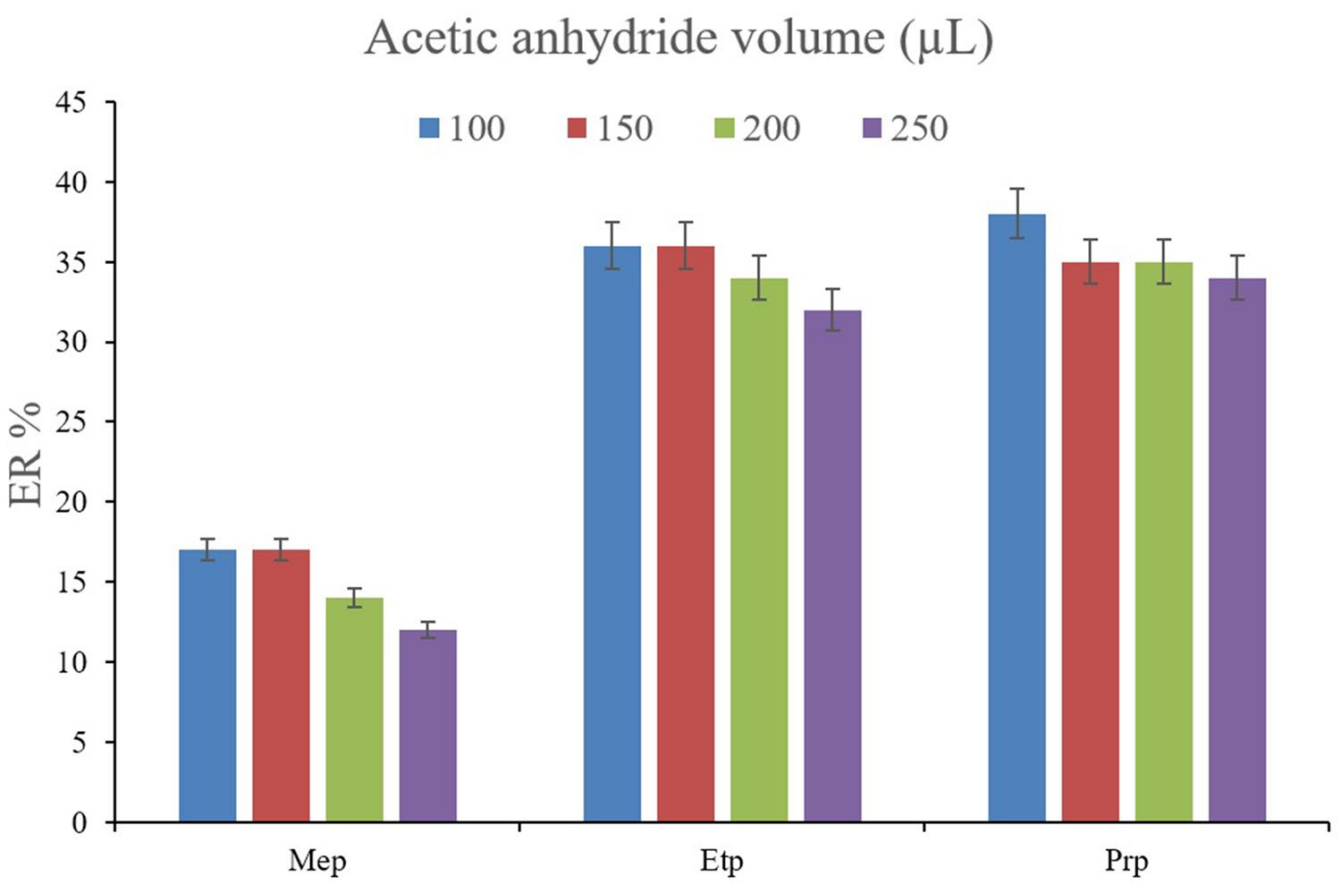
Optimization of acetic anhydride volume. Extraction conditions: are the same as those used in Fig. 2, except that derivatization was done in the pre-absorption step.

The volume of acetic anhydride as the derivatization agent was optimized in this step. For this aim, 50, 100, 150, 200, and 250 µL of acetic anhydride was added into the aqueous solution of the DµSPE step alongside the addition of 100 mg sodium carbonate. The pH values for the solutions were measured to be 8.0, 6.5, 6.1, 5.2, and 4.2, respectively. No analyte peak was seen in the case of 50 µL acetic anhydride use. It shows that the extraction does not take place in this condition. The phenomenon stems from two reasons; the first reason is the destruction of MIL-68 (Ga) which can be seen as the decomposition of absorbent particles in the basic solution by vortexing. The second reason can be related to the fact that the phenolic compounds of interest transform to their ionic forms and the incident increases their solubility in the aqueous solution. So, the extraction of the parabens in the case of 50 µL acetic anhydride use was not acceded. According to Fig. 3, all the other tested solutions resulted in the extraction of the parabens. It can be seen that increasing the derivatization agent volume over 100 µL has a slightly negative effect on the ERs. This can stem from the increase in the acidity of the solutions that can negatively impact the derivatization process. Also, using 100 µL is more economical. So, 100 µL of acetic anhydride was used as the derivatization agent in this study.

**Figure 4:**
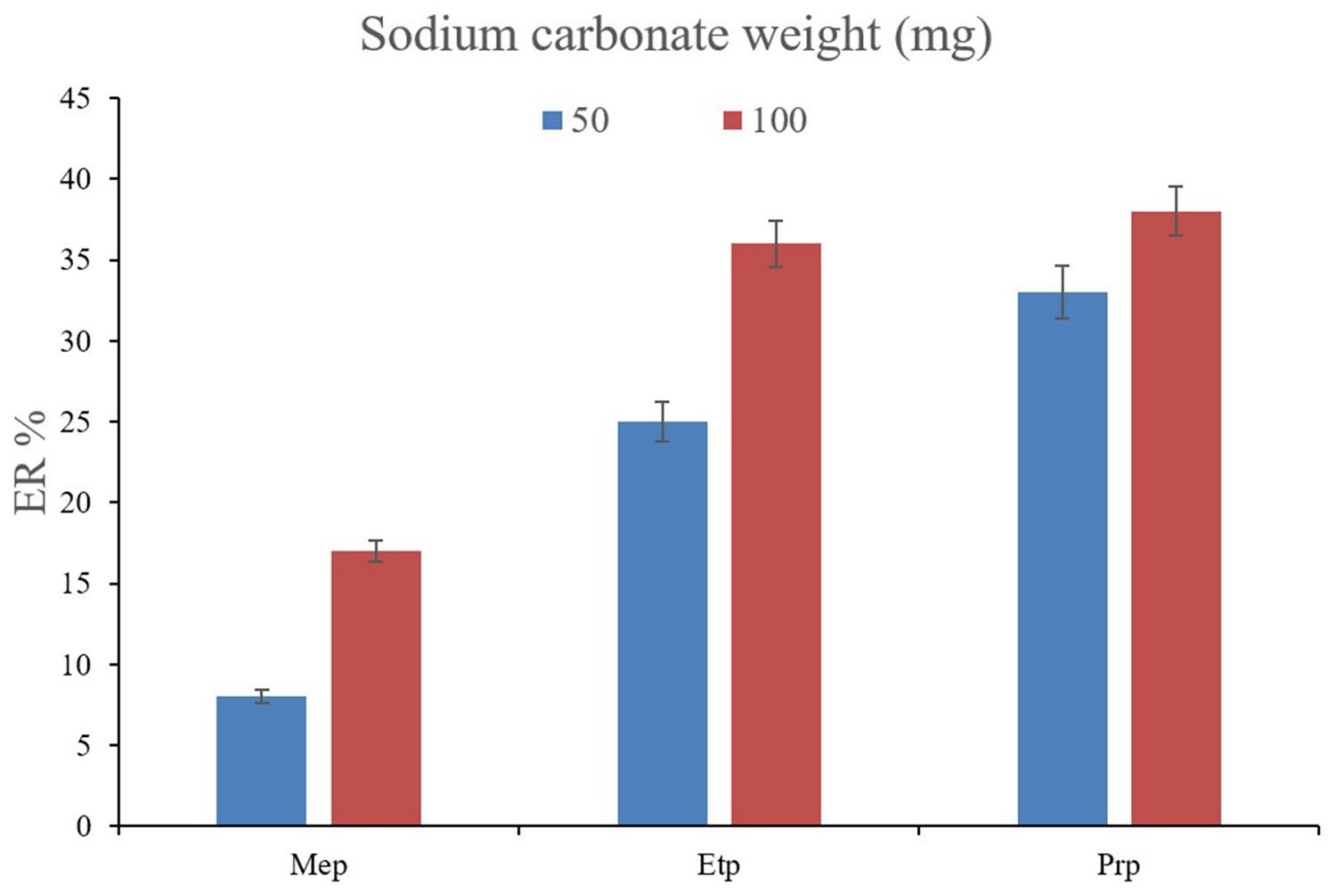
Optimization of sodium carbonate weight. Extraction conditions: are the same as those used in Fig. 3, except that 100 µL of acetic anhydride was used for derivatization.

The other important parameter that controls the derivatization procedure is the amount of sodium carbonate that paves the way for the derivatization of the surveyed parabens using acetic anhydride. To appraise the variable, 50, 100, 150, and 200 mg besides the addition of no sodium carbonate into the solution were investigated. The solution pH values were recorded as 5.1, 6.5, 7.5, 8.2, and 3.0, respectively. It was seen that in the case of the addition of no sodium carbonate, no chromatographic peak appeared. The observation can have two originations; the first is the negative absorptive effect that the acidic pH can pose to the MOF. The second reason is the absence of a derivatization process that occurs neither in DµSPE nor DLLME. The absence of derivatization in the DLLME section can lead to the participation of the analytes toward the aqueous solution which inhibits their extraction into the organic phase. Also, no chromatographic peak was detected when 150 and 200 mg sodium carbonate were used. It was vividly observed that the MOF was being decomposed when being subjected to the solutions of interest. So, the absorbent loss is the main reason for not getting analyte peaks in the related chromatograms. In Fig. 4, it can be seen that the application of 50 and 100 mg sodium carbonate leads to the extraction of the compounds of interest. The experimental data show that 100 mg sodium carbonate precedes 50 mg. This can originate from the solution pH which is slightly acidic in the case of 50 mg which negatively affects the absorptive capability of MIL-68 (Ga) and also the derivatization procedure. According to the obtained results, 100 mg of sodium carbonate was used to moderate the pH value of the solution to propel the derivatization step soundly.

**Figure 5:**
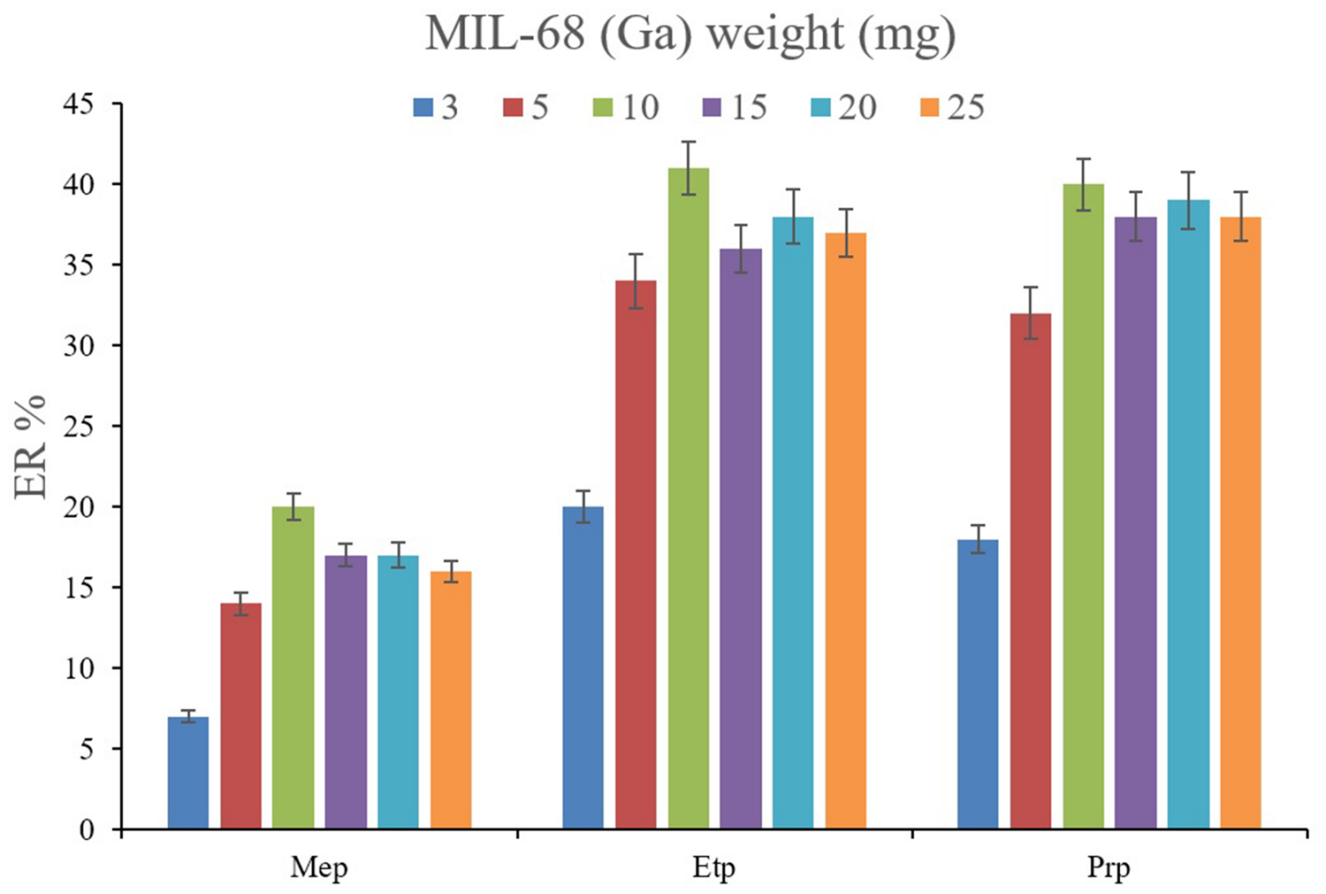
Optimization of MIL-68 (Ga) weight. Extraction conditions: are the same as those used in Fig. 4, except that 100 mg of sodium carbonate was used.

The prominent factor that controls ERs by providing sufficient surface area for the absorption aim is the weight of MIL-68 (Ga). To evaluate the efficiency of the MOF, 3, 5, 10, 15, 20, and 25 mg of MIL-68 (Ga) were subjected to the extraction of the derivatized parabens. It is seen in Fig. 5 that the use of only 10 mg of MIL-68 (Ga) is enough to reach the optimum ERs. It can be understood that the application of 3 and 5 mg of the MOF results in deficient absorption of the parabens due to the lack of sufficient surface area. Also, the use of 15, 20, and 25 mg of MIL-68 (Ga) leads to lower ER values in comparison to the use of 10 mg in the cases of all the analytes. This phenomenon originates from the agglomeration of the MOF particles that restricts the successful absorptive contacts among the analytes and MOF particles. Furthermore, lower ERs in the case of applying higher MOF weights can stem from the deficiency in the elution step using the desorption solvent. According to the results, 10 mg of MIL-68 (Ga) was chosen to be applied in every batch extraction process. This is economical and proves the high efficiency and capability of the MOF for the absorption of the surveyed polar analytes from the aqueous solution.

**Figure 6:**
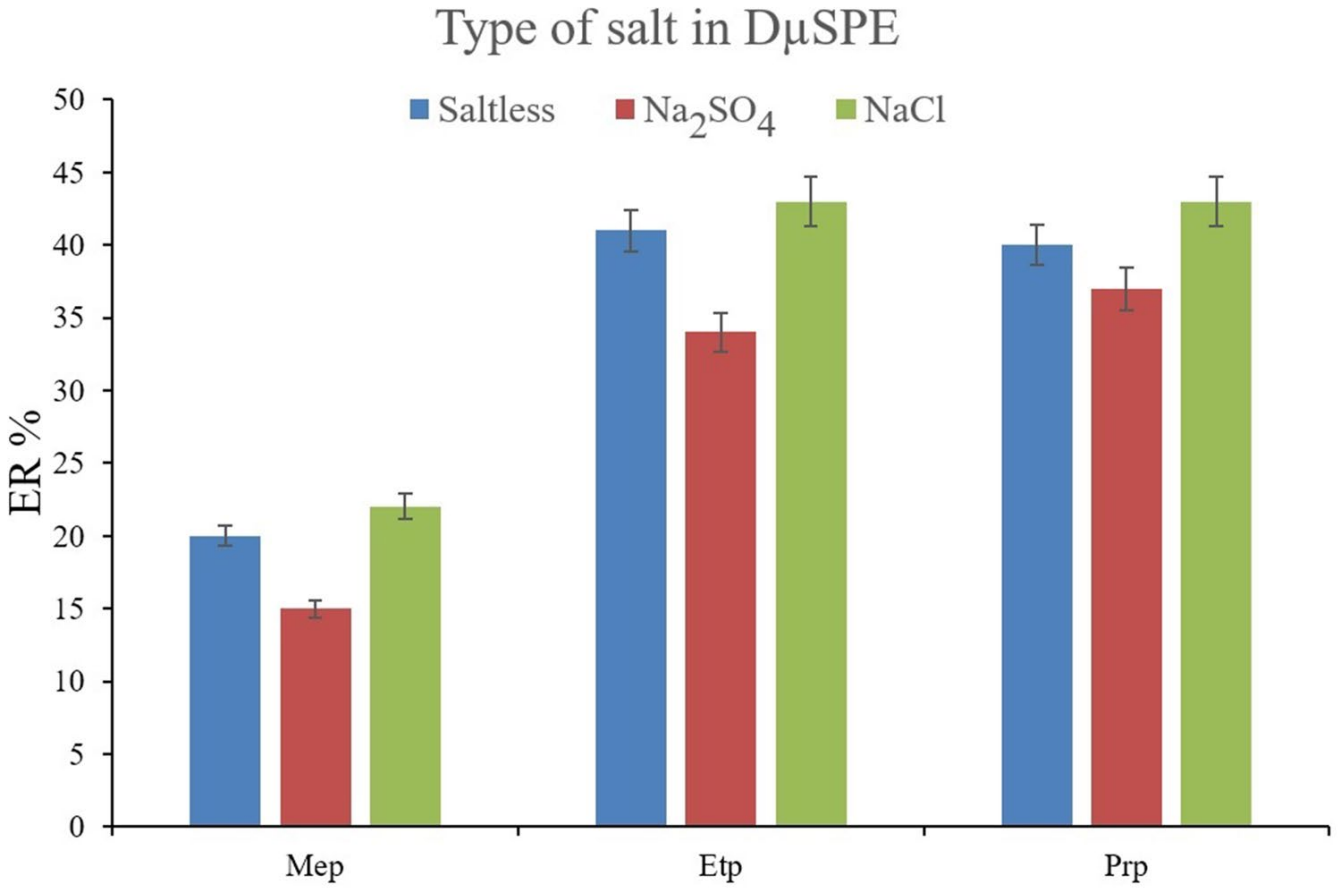
Optimization of salt type in DµSPE. Extraction conditions: are the same as those used in Fig. 5, except that 10 mg of MIL-68 (Ga) was used.

**Figure 7:**
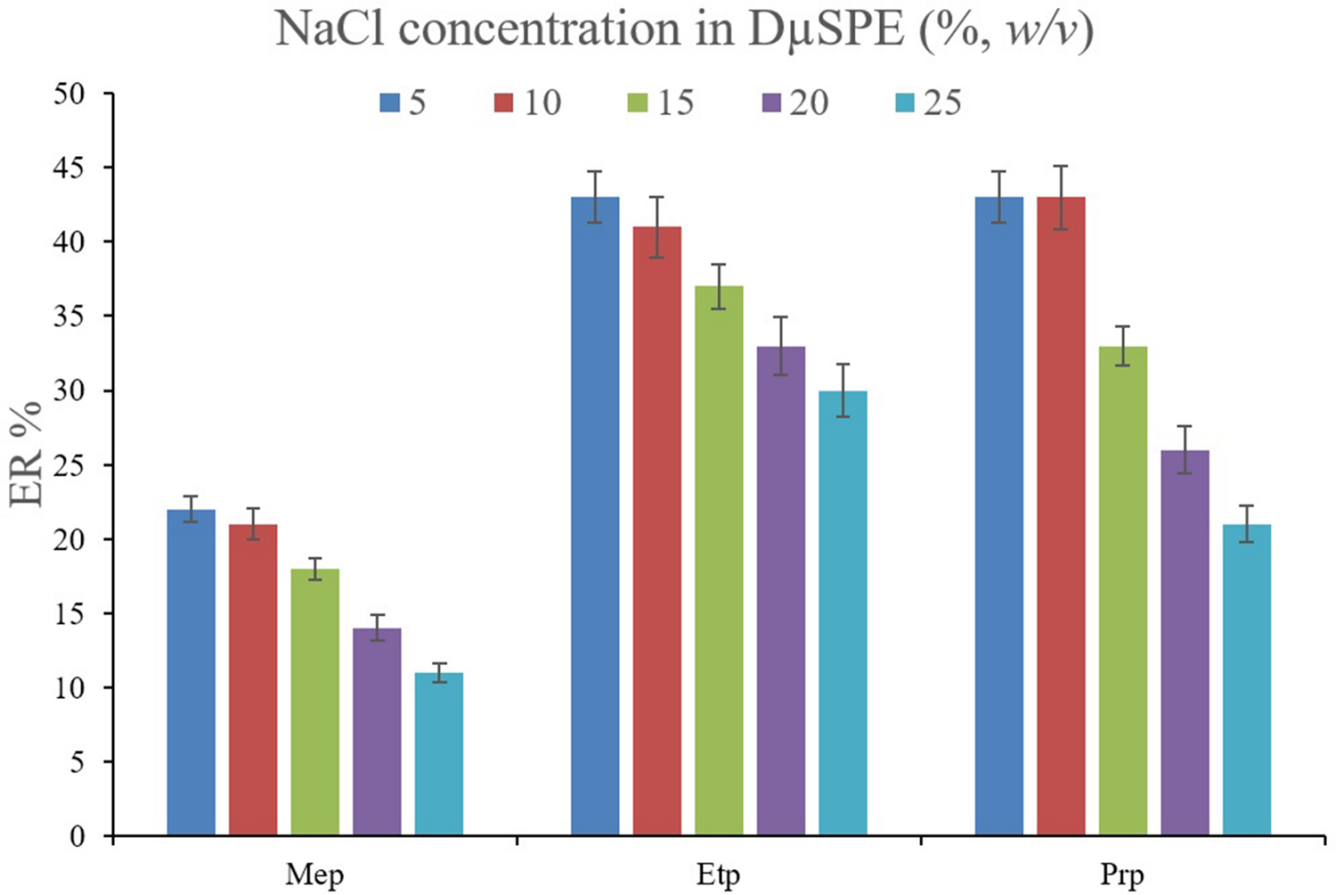
Optimization of sodium chloride concentration in DµSPE. Extraction conditions: are the same as those used in Fig. 6, except that sodium chloride was chosen as the salting-out agent.

In the case of extracting parabens, the impact of the salting-out effect can be more significant. Because parabens have high solubilities in the aqueous media, salt addition can dwindle their solubility and consequently increase their absorption by the MOF particles. To appraise this effect, solutions with 5%, *w/v* concentration of sodium chloride and sodium sulfate were prepared and subjected to the extraction of parabens alongside the saltless solution. The comparison of the obtained data is shown in Fig. 6. It is seen that the presence of sodium sulfate negatively affects the ER values. The observation can stem from the basicity of sulfate ions which enhances the pH of solutions. On the other hand, the addition of sodium chloride increases the ERs for all the surveyed parabens. So, it can be concluded that the presence of sodium chloride in the solution can efficiently act as a salting-out agent. Accordingly, the concentration of sodium chloride in the aqueous solution varied in the range of 5–25% *w/v* (with intervals of 5%). According to Fig. 7, increasing the concentration of sodium chloride leads to a reduction in the ER values. It can be concluded that higher than 5%, *w/v* concentrations of sodium chloride increase the aqueous solution's viscosity and reduce the analytes' absorption by the particles of MIL-68 (Ga).

The pH of the solution of interest can have important effects on the ER values. To evaluate this parameter, the solution pH was set at values of 2.5, 4.5, 6.5, 8.5, and 10.5. It was observed that (data not shown here) the MOF was decomposed at the basic pH values. So, the absence of the absorbent resulted in no extraction. On the other hand, the acidic pH values led to low ERs that can stem from the negative absorptive impact of acidic solutions on the MOF performance. So, no pH variation was done and the extractions were performed at the pH value of 6.5, which is the solution's pH value after adding sodium carbonate and acetic anhydride.

Vortexing properly facilitates the absorption of the surveyed parabens by the MOF. To assess the impact of vortexing time, 3, 4, 5, 6, and 7 min were implemented. The experiments showed that (data not shown here) vortexing for 5 min was sufficient to reach the optimum absorption of the analytes. So, the solutions were vortexed for 5 min.

Following the absorption of the analytes from the aqueous solution by the MOF, it is necessary to transfer them into an organic solvent called a desorption solvent. The chosen desorption solvent in this step will also act as the disperser solvent of the DLLME section. So, it has to be miscible in aqueous and organic solutions and be able to dissolve the analytes. Thus, three solvents including methanol, ACN, and 2-propanol were selected to observe their function. It is seen in Fig. S1 that methanol acts better than the two other organic solvents according to the obtained ERs. Also, using methanol leads to puffing up the MOF particles which facilitates the desorption of parabens from MIL-68 (Ga). This is observable in Fig. 1a. So, methanol was selected as the desorption solvent. In the next step, the volume of methanol was investigated using 0.5, 1.0, and 1.5 mL. Figure S2 demonstrates that using 1.0 mL of methanol is the optimum value. The results can stem from the fact that 0.5 mL of methanol is insufficient to perform the efficient desorption process. Also, 1.5 mL of methanol leads to low ERs which can be because of decreasing the polarity of the aqueous solution of DLLME that dwindles the migration of the target compounds into the extracted phase. So, 1.0 mL of methanol was adopted to be applied in the procedure.

In the desorption step, vortexing is necessary to reduce the equilibrium time to transfer the analytes from the MOF into the methanolic phase. To survey the effect of applying different vortexing times, 2, 3, 4, 5, 6, and 7 min were applied. The outcome of the experiments (data not shown here) demonstrated that 5 min vortexing was sufficient to perform the desorption step well. So, vortexing for 5 min was adopted in this step.

Adding an extraction solvent into the methanolic solution and its injection into an aqueous phase is the target of this step. The extraction solvent has to be immiscible in the aqueous phase and also create a cloudy solution during the injection of its mixture with methanol into the aqueous phase. In this study, three extraction solvents including carbon tetrachloride, 1,2-DBE, and 1,1,1-TCE were tested. 27, 32, and 34 µL of the mentioned solvents, respectively, were applied to obtain 10 ± 0.5 µL of the sedimented phase. Figure S3 shows that 1,2-DBE extracts the surveyed parabens with the highest ERs. In the next step, different volumes of 1,2-DBE including 32, 36, and 40 µL were used. Since the sedimented phase volume increased by increasing the initial volume of 1,2-DBE, the EFs dwindled (data not shown here). In fact, the dilution effect resulted in decreasing the EF values. So, 32 µL of 1,2-DBE was adopted as the extraction solvent.

Since the dispersion of the droplets of 1,2-DBE takes place in the aqueous solution of DLLME, partitioning of the analytes occurs between the aqueous and organic phases. Salt addition can be helpful to lead the partitioning toward the organic phase. Salt addition can perform the salting-out effect by decreasing the analytes’ solubility in the aqueous phase. On the other hand, increasing the salt concentration to more than the optimum value can negatively impact the ERs by viscosity enhancement of the aqueous solution of DLLME. To obtain the experimental data, sodium chloride and sodium sulfate salts (5%, *w/v,* of each) were examined and the ERs were compared with the saltless DLLME solution. Although 32 µL of 1,2-DBE was used in the case of the saltless solution, 29 µL was applied for each of the salty solutions to obtain 10 ± 0.5 µL of the sedimented phase. Figure S4 shows that sodium chloride enhances the ERs. So, the concentration of sodium chloride was altered in the range of 5, 10, and 15% *w/v*. 29, 25, and 23 µL of 1,2-DBE, respectively, were used to obtain 10 ± 0.5 µL of the sedimented phase. The obtained data in Fig. S5 shows that dissolving 10%, *w/v,* sodium chloride in the aqueous solution of DLLME increases the ERs to the optimum level in the cases of Etp and Prp. The viscosity enhancement of the solutions limits the migration of Etp and Prp into the extraction solvents in the case of 15% *w/v*. On the other hand, due to the higher solubility of Mep in the aqueous phase, 15% *w/v* of sodium chloride content results in a higher ER value that stems from the salting-out effect. By preferring Etp and Prp, 10%, *w/v,* sodium chloride was selected to be used in the DLLME section of the extraction.

It is worth mentioning that in chromatography-based research, the notion of selectivity is determined by the chromatographic system. The applied column in the GC-FID system is able to separate the surveyed parabens from the co-extractive compounds extracted from the real samples. The illustrated chromatograms in Fig. S6 vividly illustrate the successful separation of the parabens from other extracted chemicals from the matrices of the surveyed samples. Moreover, since the developed method is a sample preparation approach, it accomplishes a clean-up procedure besides performing the extraction and preconcentration aims which decreases the extraction of other chemicals from the samples and increases the selectivity boosting the function of the chromatographic system. Furthermore, the optimization procedures were carried out according to the ER enhancement of parabens. So, while optimizing the analytes’ extraction, the method was becoming more selective towards the extraction of parabens. This makes the approach more selective for the target compounds. Conclusively, the functions of the chromatographic system (which is inherently responsible for the separation aim and determines the selectivity) besides the clean-up performance of the method and the optimization of the approach for the extraction of parabens create favorable selectivity for analyzing the compounds of interest.

### Validation of the developed method

The obtained figures of merit for the developed method based on MIL-68 (Ga) are consolidated in Table 1. The table includes the data about linear range (LR), relative standard deviation (RSD), EF, ER, LOD, LOQ, and coefficient of determination (r^2^). LODs and LOQs were calculated based on signal-to-noise ratios of 3 and 10, respectively. They were calculated for the obtained analytical signals compared to their neighbor noise signals. In this case, the peak heights were used to record the signal and noise values. As can be seen, wide LRs (265–30,000 µg L^−1^) have been achieved for the parabens leading to determining their concentrations in different samples. Also, the equations of the drawn calibration curves are presented in the table. The r^2^ values are documented to be 0.999 for Etp and Prp and 0.996 for Mep. So, the method calibration curves are highly linear. The LODs and LOQs are favorable ranging from 31–80 and 106–265 µg L^−1^, respectively. The RSDs for intra- (n = 5) and inter-day (n = 4) precision values are in the ranges of 3.5–4.0 and 3.8–4.5%, respectively, for the analytes with the concentration of 1 mg L^−1^ of each. Moreover, appreciable ERs for the extraction of parabens were obtained in the range of 40–66%. It is mentioned that High EF values are noticeable ranging from 200 to 330.

**Table 1:** The figures of merit for the extraction of parabens using MIL-68 (Ga).

Analyte	LOD^a^	LOQ^b^	LR^c^	r^2d^	Calibration curve equation	RSD %^e^		EF ± SD^f^	ER ± SD^g^
						Intra-day	Inter-day		
Mep	80	265	265–30,000	0.996	y = 15297x-19653^h^	4.0	4.5	200 ± 10	40 ± 2
Etp	31	106	106–30,000	0.999	y = 32303x + 2871.8	3.5	3.8	325 ± 10	65 ± 2
Prp	76	255	255–30,000	0.999	y = 38355x-20732	3.7	4.3	330 ± 15	66 ± 3

^a^Limit of detection (S/N = 3) (µg L^−1^).

^b^Limit of quantification (S/N = 10) (µg L^−1^).

^c^Linear range (µg L^−1^).

^d^Coefficient of determination.

^e^Relative standard deviation at a concentration of 1 mg L^−1^ of each analyte for intra- (n = 5) and inter-day (n = 4) precisions.

^f^Enrichment factor ± standard deviation (n = 3).

^g^Extraction recovery ± standard deviation (n = 3).

^h^y = peak area and x = concentration (mg L^−1^).

### Analysis of real samples

Following the optimizations in the aqueous solutions, relative recovery values have to be calculated in order to subject the developed extraction method to real samples. Relative recovery is the ratio of an analyte’s peak area in a real sample to the same term in the aqueous solution (both spiked with the same concentration of the analytes) multiplied by 100. The surveyed solutions using the developed method were mouthwash and hydrating gel samples. To reduce their matrix effect, they were diluted with deionized water at 1:4 and 0.5:4.5 (*v/v*) ratios, respectively. Table 2 consolidates the relative recovery data for the surveyed samples in this study. Favorable relative recoveries stem from the proper function of MIL-68 (Ga) in the studied samples and the appropriate dilution ratios. Figure S6 demonstrates five chromatograms. They represent the direct injection of 5000 mg L^−1^ concentration of the standard solution of parabens, 2500 mg L^−1^ concentration of the standard derivatized parabens, the extracted 10 mg L^−1^ spiked aqueous solution, and the extracted mouthwash and hydrating gel samples. The betterment of the peak shapes and the enhancement of their height and area are the results of the successful derivatization procedure. Luckily, none of the target parabens were detected in the paraben-free stated samples.

**Table 2:** Study of matrix effect in the surveyed samples spiked at different concentrations.

Analyte	Mean relative recovery ± standard deviation (n = 3)					
	Mouthwash 1 (%)	Mouthwash 2 (%)	Mouthwash 3 (%)	Hydrating gel 1 (%)	Hydrating gel 2 (%)	Hydrating gel 3 (%)
All samples were spiked with each paraben at a concentration of 1 mg L^-1^						
Mep	115 ± 3	106 ± 2	111 ± 3	94 ± 4	96 ± 2	84 ± 2
Etp	85 ± 3	91 ± 1	84 ± 2	88 ± 3	87 ± 2	85 ± 3
Prp	88 ± 2	94 ± 3	89 ± 2	100 ± 3	93 ± 4	87 ± 3
All samples were spiked with each paraben at a concentration of 5 mg L^-1^						
Mep	109 ± 2	105 ± 3	116 ± 2	96 ± 3	93 ± 4	88 ± 2
Etp	87 ± 2	93 ± 2	87 ± 3	90 ± 3	90 ± 3	89 ± 2
Prp	86 ± 3	90 ± 1	91 ± 2	98 ± 2	87 ± 2	91 ± 3
All samples were spiked with each paraben at a concentration of 10 mg L^-1^						
Mep	105 ± 3	103 ± 3	109 ± 3	101 ± 2	95 ± 3	93 ± 4
Etp	90 ± 4	96 ± 3	94 ± 3	97 ± 3	89 ± 2	95 ± 2
Prp	84 ± 3	97 ± 2	96 ± 4	103 ± 2	95 ± 4	94 ± 3

### Comparison of the method with some other approaches

To provide comprehensive data about the extraction of parabens from different matrices using various methods and also to cater comparative information on the obtained figures of merit, Table 3 was prepared. Different figures of merit including EF, ER, RSD, LOD, LOQ, r^2^, and LR values are comparatively illustrated in the table. In order to convey comprehensive data, ten previously developed methods were compared with the developed method in this study. As can be seen, various samples including swimming pool and sea water, lidocaine gel, skin tonic, mouthwash, hydrating gel, lotions, bottled water, sunblock cream, toothpaste, and toilet water have been studied for the extraction and preconcentration of their paraben content. The LRs of this study are wider than the LRs of most of the developed methods. Also, the r^2^ values are superior to most of the compared methods. The RSDs are lower than or comparable with the obtained RSD values for the compared approaches. The EFs are significantly higher than the obtained EFs in the mentioned methods. Higher than or comparable ER values have also been documented for the introduced method. Since mass spectrometer (MS) is inherently more selective and sensitive than FID, the obtained LODs and LOQs in the related cases are lower. The highlighted characteristics of the developed method are not only attributed to MIL-68 (Ga) but also influenced by the method. But, the high efficiency of the MOF is inevitable in this study. Some beneficial traits of the MOF such as appreciable surface area and pore volume, angstrom-scale mean pore diameter, containing inorganic and organic sections that provide successful intermolecular interactions with parabens, its well-dispersion in the aqueous medium for the absorption aim, puffing up in dealing with methanol which streamlines the desorption of analytes, and its small particle size are the highlighted aspects that lead to using only 10 mg of MIL-68 (Ga) to propel the extraction procedure. Moreover, performing the DLLME step provides reaching low LODs and LOQs besides obtaining high EFs.

**Table 3:** Comparison of the developed method with some similar methods.

Method	Analyte	Sample	LOD^a^	LOQ^b^	LR^c^	r^2d^	RSD (%)^e^	EF^f^	ER (%)^g^	Refs.
MCHNME-GC-MS^h^	Mep	Swimming pool and seawater	0.0861		0.5–500	0.998	7.1	–	–	^50^
	Etp		0.0346		0.1–500	0.996	6.9			
	Prp		0.0238		0.1–500	0.999	5.1			
GFA-DLPME-GC-FID^i^	Mep	Lidocaine gel, skin tonic, Aloe Vera gel, and mouthwash	1.44	4.8	5–10000	0.999	3.7	–	–	^51^
	Etp		1.63	5.44	5–10000	0.998	3.8			
	Prp		1.15	3.65	5–10,000	0.999	2.46			
MIP-SPME-GC-FID ^j^	Mep	Soy samples	0.25	0.82	10–10,000	0.9943	3.93	–	–	^52^
	Etp		0.22	0.72	10–10,000	0.9992	2.98			
	Prp		0.3	0.99	10–10,000	0.9989	3.13			
PTFF-SPME-LC-DAD^k^	Mep	Creams and lotions	120	400	500–160,000	0.996	3.3	–	–	^53^
	Etp		140	470	500–160000	0.992	5.4			
	Prp		150	500	500–160,000	0.996	3.4			
MMCNMOF-DmµSPE-HPLC-DAD^l^	Mep	Bottled water, tap water, skin cream,	0.15	–	0.5–1500	0.9996	4.4	67.9	38	^54^
		foot cream and sunblock cream								
SPGMN-MSPE-GC-FID^m^	Mep	Sunscreen cream, moisturizing cream, and toothpaste	2.8	–	Oct-00	0.99	7.9	190	–	^55^
	Prp		1.5		May-00	0.997	6.3	289		
SALLME-GC-FID^n^	Mep	Tap and river water, cream, and lipstick	0.5	–	Feb-00	0.998	8.1	128	51	^15^
	Etp		1		May-00	0.997	7.6	137	55	
	Prp		0.5		Feb-00	0.996	7.8	147	59	
DLLME-GC-FID^o^	Mep	Lotion, mouthwash, and toilet water	15	–	0.02–10.0	0.9997	3.3	87	–	^56^
	Etp		4.8		0.025–5.0	0.9986	5.2	178		
	Prp		6.3		0.025–5.0	0.9996	4.2	187		
RDSE-GC-MS^p^	Mep	Tap water	0.02	0.06	–	0.998	2.5	–	79.5	^57^
	Etp		0.02	0.08		0.999	2.8		82.1	
	Prp		0.04	0.12		0.998	9.7		82.1	
SBSE-GC-MS^q^	Mep	Tap water and wastewater	0.00186	0.00617	–	0.9999	5.9	–	–	^58^
DES-VALLME-HPLC-UV ^r^	Mep	Mouthwash	6.1	20.3	10–25,000	0.999	1.41	–	–	^59^
	Etp		5.3	17.6	10–25,000	0.999	1.22			
	Prp		4.6	15.3	10–25,000	0.999	0.7			
EAE-HPLC-UV^s^	Mep	Pharmaceuticals	25.7	51.4	50–510	0.999	1.5	–	–	^60^
	Etp		25.2	50.4	50–500	0.999	0.5			
	Prp		25.9	51.8	50–520	0.999	0.5			
DµSPE-DLLME-GC-FID^t^	Mep	Mouthwash and hydrating gel	80	265	265–30,000	0.996	4	200	40	Present method
	Etp		31	106	106–30,000	0.999	3.5	325	65	
	Prp		76	255	255–30,000	0.999	3.7	330	66	

^a^Limit of detection (µg L^−1^).

^b^Limit of quantification (µg L^−1^).

^c^Linear range (µg L^−1^).

^d^Coefficient of determination.

^e^Relative standard deviation.

^f^Enrichment factor.

^g^Extraction recovery.

^h^Magnetically confined hydrophobic nanoparticles microextraction-gas chromatography-mass spectrometry.

^i^Gas flow-assisted dispersive liquid-phase microextraction-gas chromatography-flame ionization detector.

^j^Molecularly imprinted polymer-based solid phase microextraction-gas chromatography-flame ionization detector.

^k^Poly(ethylene glycol) diacrylate thin film fibers-based solid phase microextraction-liquid chromatography-diode array detector.

^l^Magnetite multiwalled carbon nanotubes/metal–organic framework-based dispersive magnetic micro solid phase extraction-high performance liquid chromatography-diode array detector.

^m^Self-doped polyaniline immobilized on the graphene-modified magnetite nanoparticles-based magnetic solid phase extraction-gas chromatography-flame ionization detector.

^n^Syringe-assisted liquid–liquid microextraction-gas chromatography-flame ionization detector.

^o^Dispersive liquid–liquid microextraction-gas chromatography-flame ionization detector.

^p^Rotating disk sorptive extraction-gas chromatography-mass spectrometry.

^q^Stir bar sorptive extraction-gas chromatography-mass spectrometry.

^r^Deep eutectic solvent-based vortex assisted liquid–liquid microextraction-high performance liquid chromatography-ultraviolet detector.

^s^Effervescence-assisted extraction-high performance liquid chromatography-ultraviolet detector.

^t^Dispersive micro solid phase extraction-dispersive liquid–liquid microextraction-gas chromatography-flame ionization detector.

### The extraction mechanism of parabens using MIL-68 (Ga)

Due to the fact that the solubility of parabens in the aqueous solution is significant, their successful extraction using a micro solid phase extraction procedure is noteworthy. So, the evaluation of the absorption mechanism is interesting. According to the structures of the surveyed parabens and MIL-68 (Ga), it can be concluded that π–π stacking plays an important role in the adsorption of the analytes onto the MOF. Hydrogen bonds are also created among the oxygen-linked hydrogens in the non-derivatized parabens and oxygen atoms of the MOF’s ligand. The notion can be a superiority for the adsorption of the non-derivatized parabens onto MIL-68 (Ga). Moreover, polar-polar intermolecular interactions can take place among the oxygen atoms of the analytes and the MOF. Actually, it has to be added that the overall similarity among the structures of the analytes and the ligand section of the MOF leads to overlapping among them that eventually results in successful adsorption. Figure S7 schematically demonstrates the derivatized and non-derivatized parabens and their conversion procedure to picture the discussed interactions visually. From another point of view, BET analysis revealed a 23.517 Å average pore diameter for the synthesized MOF. Thus, it can be understood that the MOF benefits from a semi-microporous advantage since the pores are proportional to the size of some organic molecules. In the case of this study, the molecular approximate longitudinal and transverse proportions of Mep, Etp, and Prp were calculated using Avogadro software (version 1.2.0) following the optimization of the molecules’ geometry^48^. The blue arrows in the figure denote the calculated distances (D). The outcome of the calculations is shown in Fig. S8. By comparing the average pore diameter of MIL-68 (Ga) (23.517 Å) and the longitudinal and transverse proportions of the derivatized and non-derivatized parabens (8.835–12.391 and 4.323–7.829 Å, respectively), it can be concluded that both types of the analytes can enter into the provided pores of the MOF. So, the analytes can be trapped in the MOF pores. This can be a promising hint to be added to the discussed intermolecular interactions to prove the successful extraction of parabens from the aqueous solution by the MOF.

### Application of complementary green analytical procedure index (ComplexGAPI)

To provide an all-embracing assessment of the greenness feature of the developed method ComplexGAPI software was used^49^. Three colors including green, yellow, and red are used to develop the related pictogram of the analytical method. They appear from high to low compatibility of the method with the principles of green chemistry. Figure S9 demonstrates the obtained pictogram for the developed MIL-68 (Ga)-based method. The need for no special preservation and transportation steps for the samples, low amount use of solvents and the MOF, no emission of hazardous vapors into the atmosphere, the ability to perform qualitative and quantitative analysis, compatibility with the green economy rules, low health and safety hazards, and simple workup section are the highlights of the developed method.

## Conclusions

For the first time in this research, MIL-68 (Ga) was used to develop an analytical method. The method was successfully applied for the extraction of parabens from mouthwash and hydrating gel samples. The capability of the MOF for the absorption of both derivatized and non-derivatized parabens was appreciable. The mechanism was discussed according to intermolecular interactions and Avogadro software results. ComplexGAPI software was applied to investigate the greenness of the developed method from different points of view. The derivatization process increased the peak area and height, and prevented peak tailing. Performing the derivatization process amidst the DµSPE procedure imposes no additional step or effort for the method. So, high EFs (200–330) were achieved. Also, it decreased the solubility of the analytes which resulted in reaching reasonable ERs (40–66%). Low MOF use (10 mg) in the batch analysis and low temperature and short time for the synthesis made the approach economical. Puffing up MIL-68 (Ga) particles after being treated with methanol helped to desorb the analytes well. Wide LRs (265–30,000 µg L^−1^) enabled the measurement of the target compounds in a wide range of concentrations. Appreciable r^2^ values (0.996–0.999) denote the linearity of the calibration curves. The LODs and LOQs were favorable ranging from 31 to 80 and 106–265 µg L^−1^, respectively. The RSDs for intra- (n = 5) and inter-day (n = 4) precision values were in the ranges of 3.5–4.0 and 3.8–4.5%, respectively, for the analytes with the concentration of 1 mg L^−1^ of each. The capability of MIL-68 (Ga) and the favorable figures of merit obtained for the developed method led to the successful extraction and preconcentration of Mep, Etp, and Prp from mouthwash and hydrating gel samples. Actually, there are some potential improvements that can be accomplished in further research. The applied separation and detection system in this study is GC-FID which can be improved to GC–MS that is more selective and sensitive. The conversion can potentially lead to comparatively lower LODs and LOQs. Also, the applicability of MIL-68 (Ga) for the extraction and preconcentration of other probable analytes can be surveyed. The probability of producing a magnetic composite with the MOF can be assessed for the development of magnetic dispersive micro solid phase extraction to eliminate centrifugation steps. Furthermore, the probability of the synthesis of MIL-68 (Ga) in the aqueous solution instead of using DMF can be investigated to make the approach greener and safer.

### Supplementary Information

Supplementary Figures.

## Data Availability

All data generated or analyzed during this study are included in this published article.

## Abbreviations

DLLME: Dispersive liquid–liquid microextraction
DµSPE: Dispersive micro solid phase extraction
EF: Enrichment factor
FID: Flame ionization detector
GC: Gas chromatography
LOD: Limit of detection
LOQ: Limit of quantification
LR: Linear range
RSD: Relative standard deviation
Mep: Methylparaben
Etp: Ethylparaben
Prp: Propylparaben
