# Supplementary material for: MIL-68 (Ga) for the extraction of derivatized and non-derivatized parabens from healthcare products
Source: Sci Rep. 2023 Dec 2;13:21304. doi: 10.1038/s41598-023-48880-1 (PMC10693546; doi:10.1038/s41598-023-48880-1)
Supplement: Supplementary file 1 — Supplementary Figures. [file 41598_2023_48880_MOESM1_ESM.docx]

**MIL-68 (Ga) for the extraction of derivatized and non-derivatized parabens from healthcare products**

Sakha Pezhhanfar ^a^, Mir Ali Farajzadeh ^a, b*^, Mahdi Kheirkhah Ghaleh ^a^, Seyed Abolfazl Hosseini-Yazdi ^c^, Mohammad Reza Afshar Mogaddam ^d, e^

^a^ Department of Analytical Chemistry, Faculty of Chemistry, University of Tabriz, Tabriz, Iran

^b^ Engineering Faculty, Near East University, 99138 Nicosia, North Cyprus, Mersin 10, Turkey

^c^ Department of Inorganic Chemistry, Faculty of Chemistry, University of Tabriz, Tabriz, Iran

^d^ Food and Drug Safety Research Center, Tabriz University of Medical Sciences, Tabriz, Iran

^e^ Pharmaceutical Analysis Research Center, Tabriz University of Medical Sciences, Tabriz, Iran

*Corresponding author: M.A. Farajzadeh

Tel.: +98 41 33393084

Fax: +98 41 33340191

E-mail address: mafarajzadeh@yahoo.com; [mafarajzadeh@tabrizu.ac.ir](mailto:mafarajzadeh@tabrizu.ac.ir)


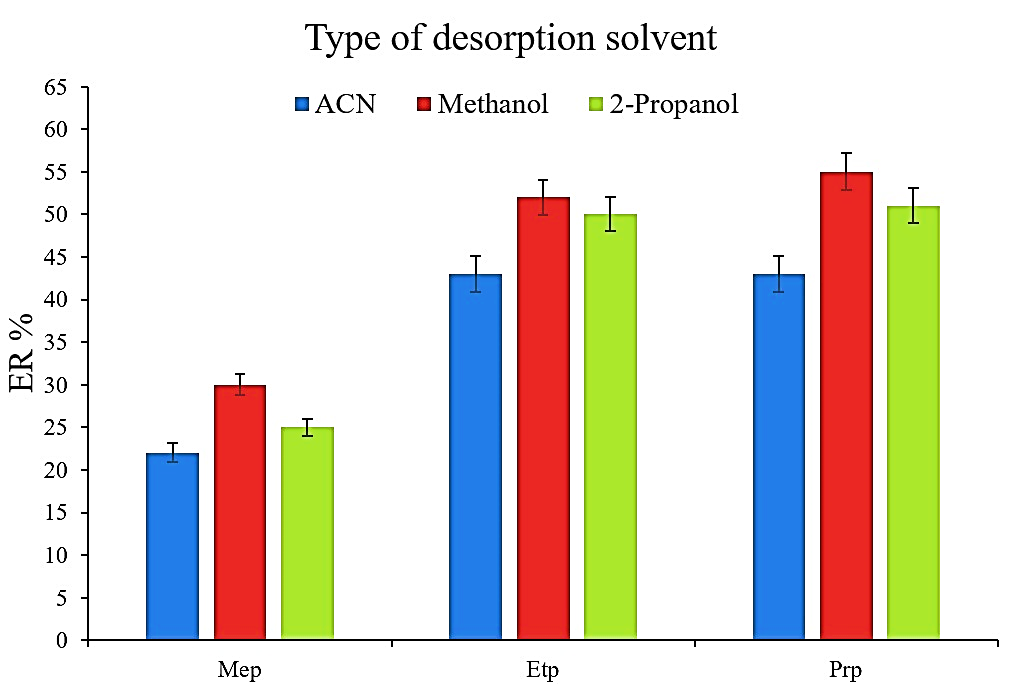


Fig. S1. Optimization of desorption solvent type.

Extraction conditions: are the same as those used in Fig. 7, except that 5%, *w/v*, sodium chloride was used.


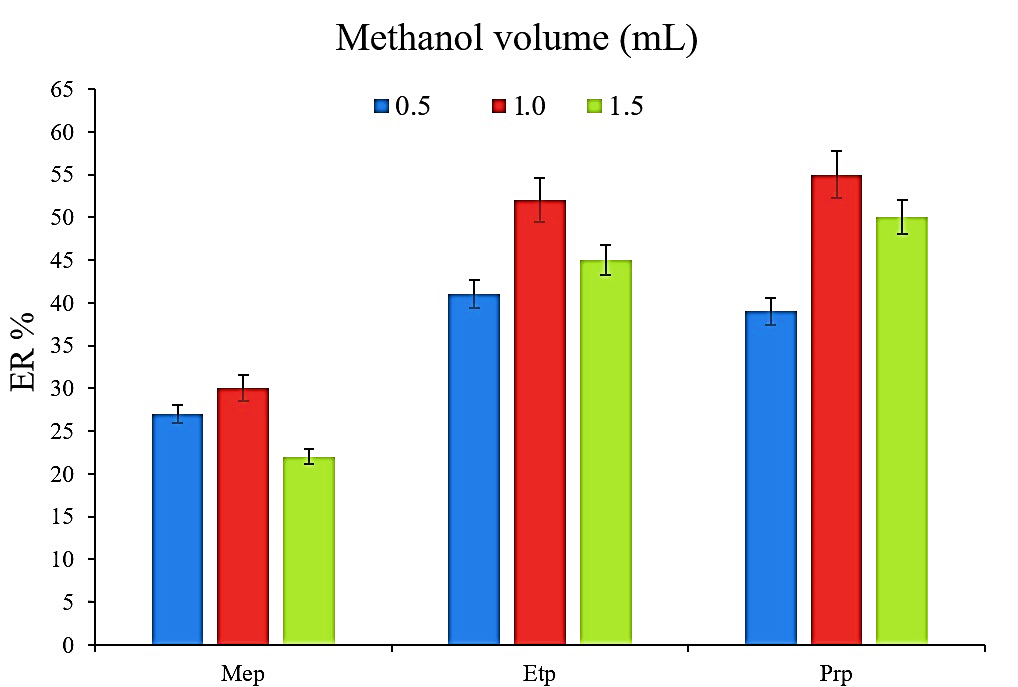


Fig. S2. Optimization of methanol volume.

Extraction conditions: are the same as those used in Fig. S1, except that methanol was selected as the desorption solvent.


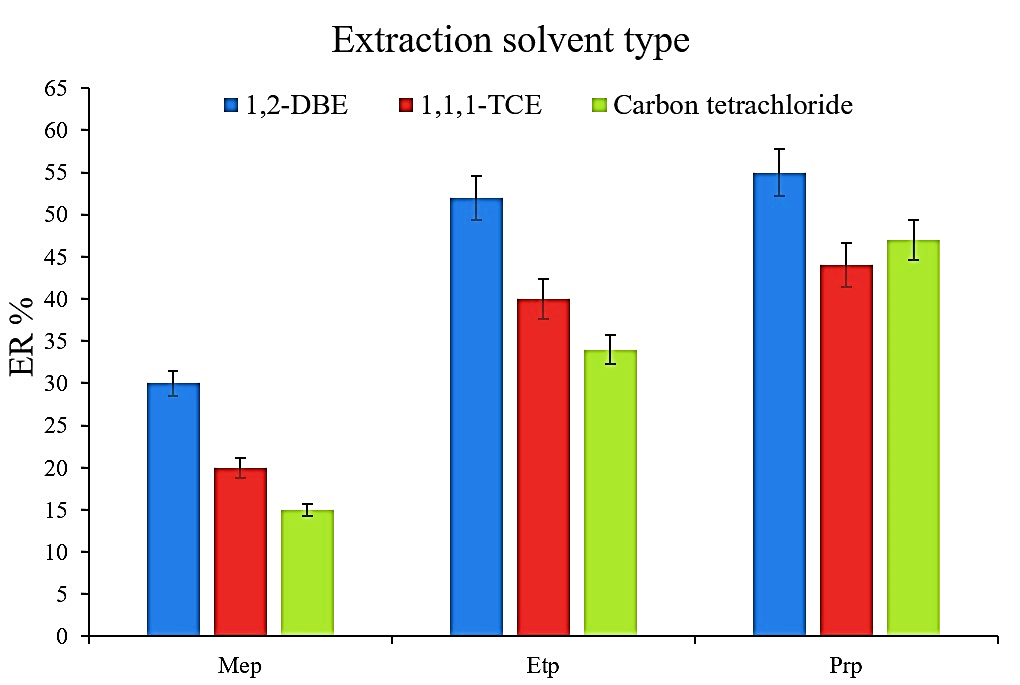


Fig. S3. Selection of extraction solvent type.

Extraction conditions: are the same as those used in Fig. S2, except that 1.0 mL methanol was selected.


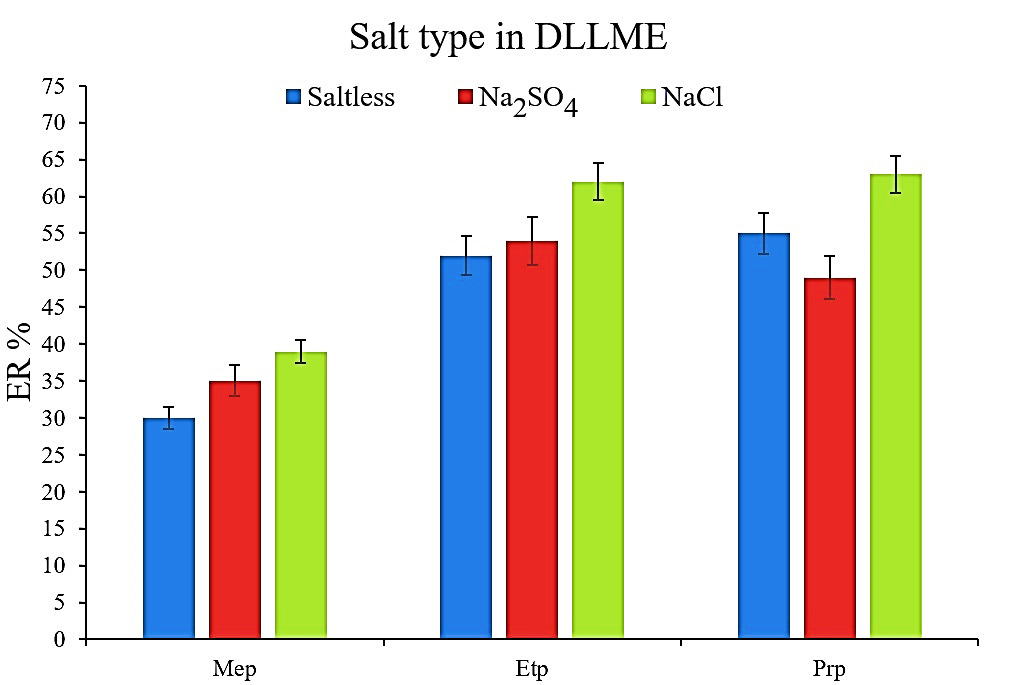


Fig. S4. Selection of salt type in DLLME.

Extraction conditions: are the same as those used in Fig. S3, except that 1,2-DBE was selected as the extraction solvent.


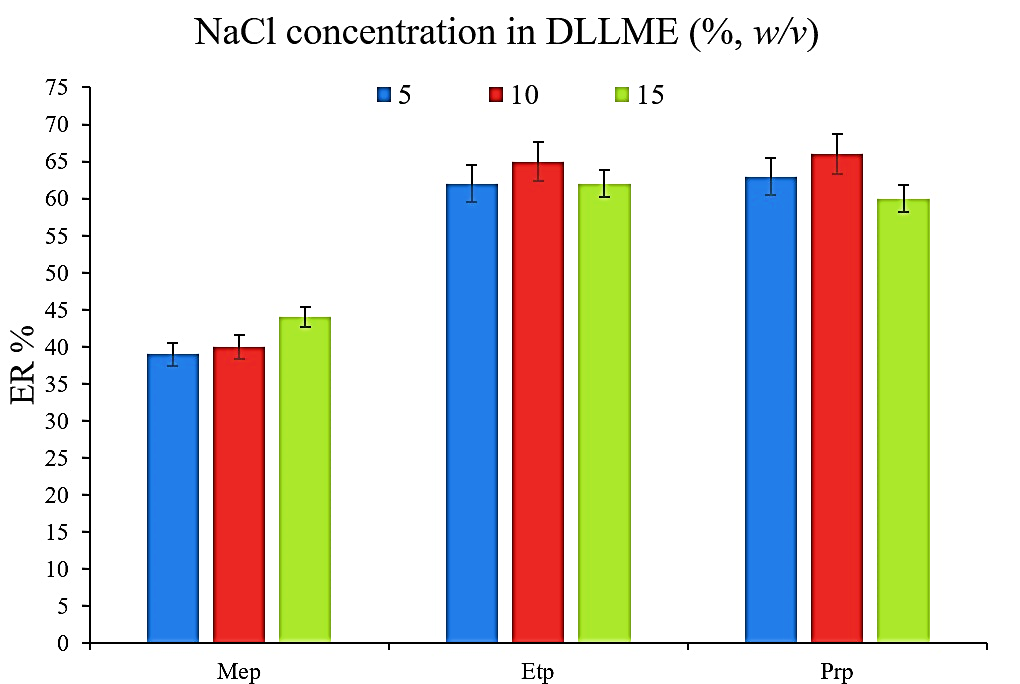


Fig. S5. Optimization of sodium chloride concentration in DLLME.

Extraction conditions: are the same as those used in Fig. S4, except that sodium chloride was selected as the salting-out agent.


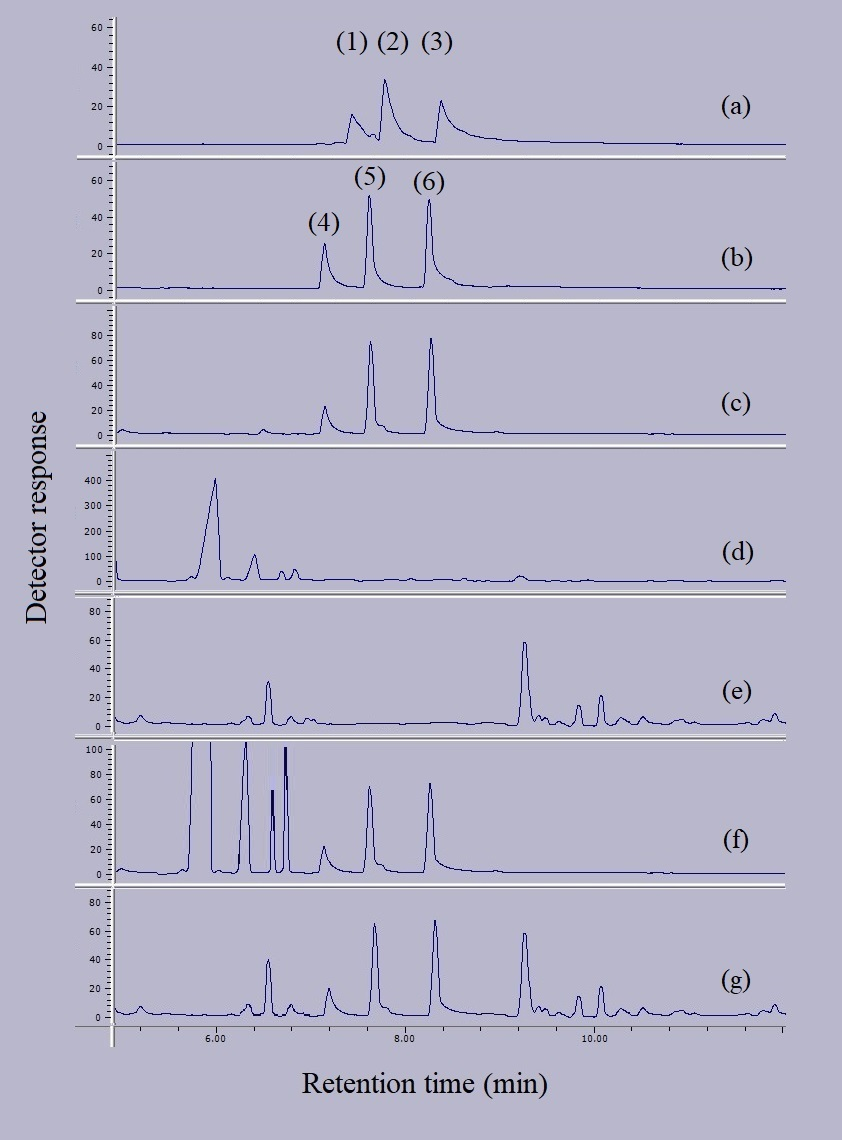


Fig. S6. Typical GC-FID chromatograms of: (a) standard solution (5000 mg L^-1^ of each paraben in methanol), (b) derivatized standard solution using acetic anhydride (2500 mg L^-1^ of each paraben), (c) deionized water spiked at a concentration of 10 mg L^-1^ of each paraben, (d) mouthwash sample, (e) hydrating gel sample, (f) mouthwash sample spiked at a concentration of 10 mg L^-1^ of each paraben, and (g) hydrating gel sample spiked at a concentration of 10 mg L^-1^ of each paraben. Except for chromatograms (a) and (b) in which direct injection without preconcentration was done, the other chromatograms were the outcome of extraction using the developed method. Peaks identification: (1) Mep, (2) Etp, (3) Prp, (4) derivatized Mep (5) derivatized Etp (6) derivatized Prp.


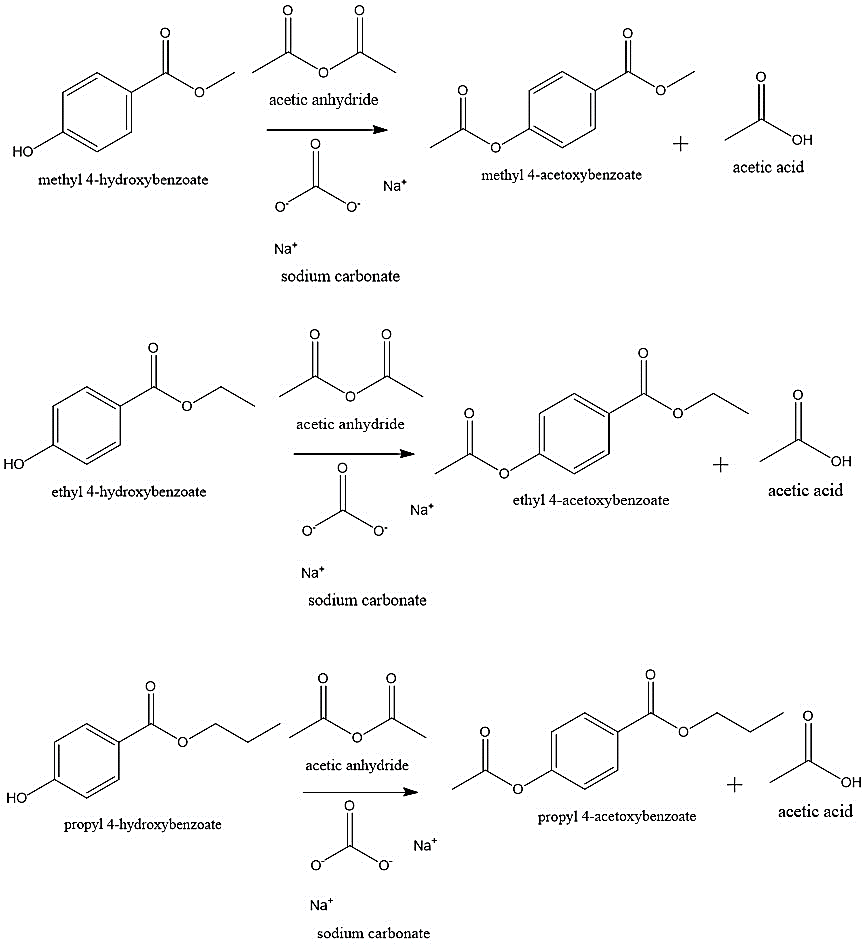


Fig. S7. The derivatized and non-derivatized parabens and their conversion procedure.


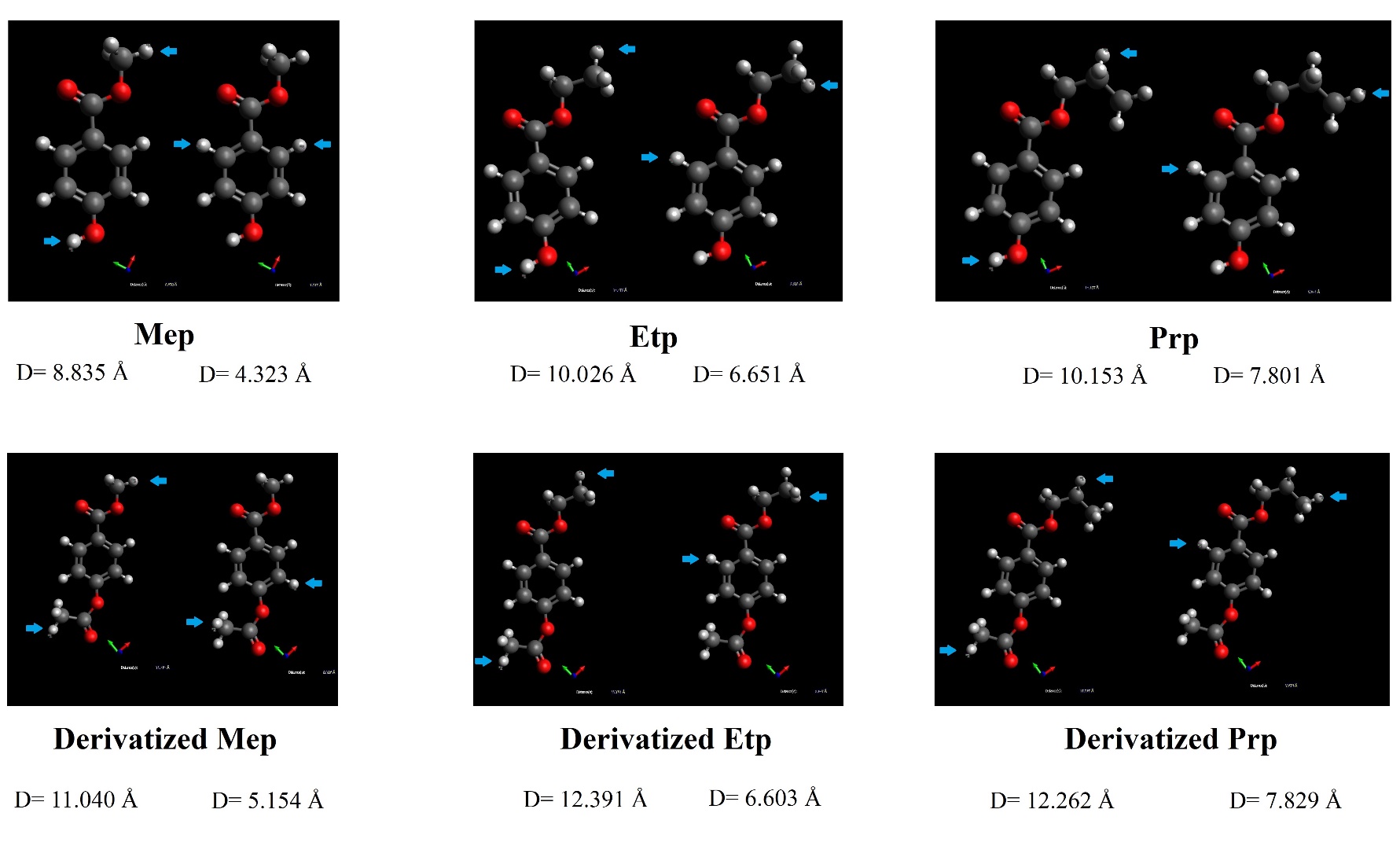


Fig. S8. The approximate longitudinal and transverse dimensions calculated by Avogadro.


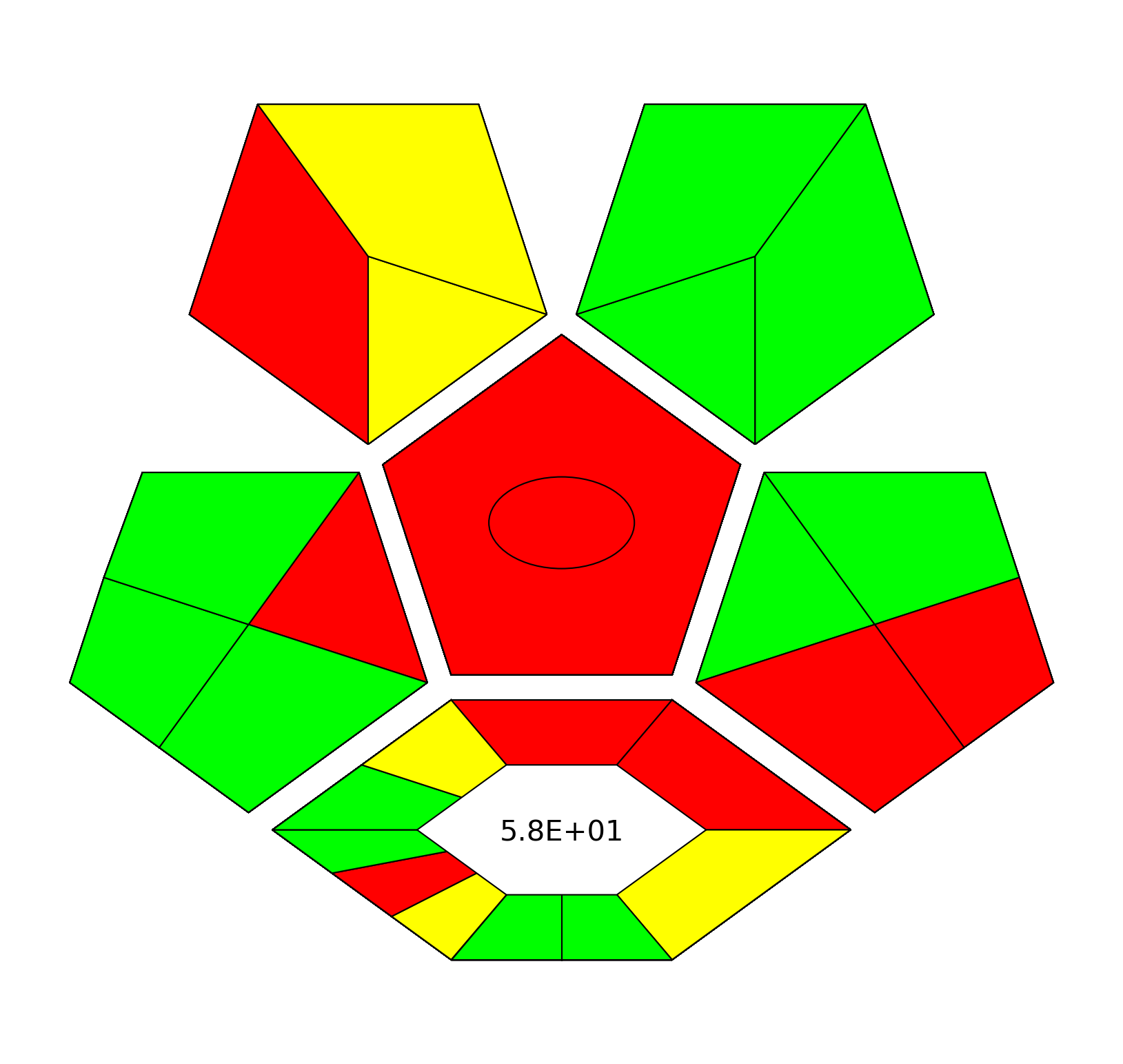


Fig. S9. The obtained ComplexGAPI pictogram for the developed method.
